# How can chiropractic become a respected mainstream profession? The example of podiatry

**DOI:** 10.1186/1746-1340-16-10

**Published:** 2008-08-29

**Authors:** Donald R Murphy, Michael J Schneider, David R Seaman, Stephen M Perle, Craig F Nelson

**Affiliations:** 1 Rhode Island Spine Center Pawtucket, RI, USA; 2 Department of Community Health, Warren Alpert Medical School of Brown University, Providence, RI, USA; 3 Department of Research, New York Chiropractic College, Seneca Falls, NY, USA; 4 Private practice of chiropractic, Pittsburgh, PA, USA; 5 School of Health and Rehabilitation Sciences, University of Pittsburgh, Pittsburgh, PA, USA; 6 Palmer College of Chiropractic, Florida Port Orange, FL, USA; 7 University of Bridgeport College of Chiropractic, Bridgeport, CT, USA; 8 American Specialty Health, San Diego, CA, USA

## Abstract

**Background:**

The chiropractic profession has succeeded to remain in existence for over 110 years despite the fact that many other professions which had their start at around the same time as chiropractic have disappeared. Despite chiropractic's longevity, the profession has not succeeded in establishing cultural authority and respect within mainstream society, and its market share is dwindling. In the meantime, the podiatric medical profession, during approximately the same time period, has been far more successful in developing itself into a respected profession that is well integrated into mainstream health care and society.

**Objective:**

To present a perspective on the current state of the chiropractic profession and to make recommendations as to how the profession can look to the podiatric medical profession as a model for how a non-allopathic healthcare profession can establish mainstream integration and cultural authority.

**Discussion:**

There are several key areas in which the podiatric medical profession has succeeded and in which the chiropractic profession has not. The authors contend that it is in these key areas that changes must be made in order for our profession to overcome its shrinking market share and its present low status amongst healthcare professions. These areas include public health, education, identity and professionalism.

**Conclusion:**

The chiropractic profession has great promise in terms of its potential contribution to society and the potential for its members to realize the benefits that come from being involved in a mainstream, respected and highly utilized professional group. However, there are several changes that must be made within the profession if it is going to fulfill this promise. Several lessons can be learned from the podiatric medical profession in this effort.

## Introduction

The chiropractic profession has been in existence for over 110 years. In that time it has overcome a variety of hardships and adversities, including practitioners being jailed for practicing medicine without a license, attempts by the American Medical Association to contain and eliminate the profession, and general ostracism by many within and outside health care [1]. It has made some remarkable advances in recent years including substantial Federal funding of chiropractic research by the National Institutes of Health and the inclusion of chiropractic physicians in the Veterans Administration healthcare system. However, in spite of this, the profession has not gained a level credibility and cultural authority in mainstream society that is required to establish itself on equal ground with other healthcare professions. The profession still finds itself in a situation in which it is rated dead last amongst healthcare professions with regard to ethics and honesty [2], and in which only 7.5% of the population utilizes its services [3], this percentage having dwindled from 10% only a short time ago [3,4].

Why have chiropractors not been able to establish themselves as a well-respected, highly utilized group of professionals who are widely seen by the public as offering essential services to society? Is it possible that the chiropractic profession can overcome its troubled past to become a mainstream, respected, highly utilized profession with an abundance of cultural authority? We believe so, and will point to the podiatric medical profession as an illustration of how the chiropractic profession could have established itself in mainstream health care, and perhaps still can.

### The Example of Podiatry

Interestingly, the podiatric medical profession has been in existence in the United States (US) for about the same amount of time as chiropractic; the first licensing laws for podiatric physicians were enacted in 1895 [5]. In the US, podiatry grew up and matured as a new profession within the same healthcare environment as chiropractic, during a time when new professions (e.g., osteopathy, homeopathy, Thompsonism) were arising out of the failure of pre-Flexner allopathic medicine to provide beneficial care for a variety of human complaints [6]. Yet, podiatrists currently find themselves far more established and respected in mainstream health care and society than chiropractors. According to the American Podiatric Medical Association (details can be found at ; accessed 29 May, 2008) many, perhaps most, major hospitals provide podiatry services, podiatrists regularly serve on the staffs of long-term care facilities, are included on the faculties of schools of medicine, serve as commissioned officers in the Armed Forces, in the US Public Health Service and in many municipal health departments.

We suggest the chiropractic profession consider several questions that speak to the different histories of the chiropractic and podiatric profession. Why are podiatrists better integrated into hospitals [7,8] and other multidisciplinary facilities [9,10] than chiropractors? Why are most schools of podiatry integrated into the university system, while chiropractic schools (with very few exceptions) are not? Why did the AMA not try to "contain and eliminate" the podiatric medical profession (despite the several turf battles podiatry has had with the orthopedic specialty)? Why were podiatrists not thrown in jail in the early days for practicing medicine without a license? How did podiatrists gain the level of cultural authority that they currently enjoy, despite having the same duration of existence and a smaller number of practitioners than chiropractic?

In the remainder of the paper we will address several key points regarding the professional attitudes and behaviors that permitted the podiatric profession to successfully mature. We feel that there are significant lessons to be learned from podiatry's successes, and that a critical look at our profession can help us to correct our mistakes and move ourselves in the direction of cultural authority, widespread acceptance, public confidence, and wide utilization.

#### 1. Public Health

One important reason podiatry succeeded in establishing itself in mainstream health care was its traditional dedication to public health [11-14]. Podiatrists became active members of the American Public Health Association (APHA) as far back as the 1950's, embracing and contributing to the advancement of accepted public health initiatives, in cooperation with others involved in public health. Podiatrists slowly gained an image as proponents of public health, at a time when many chiropractors aggressively (and dogmatically, without evidence [15]) opposed many public health measures such as vaccination and water fluoridation. As a result, podiatrists became influential members of the healthcare community, and foot health became widely recognized as an important component to overall human health.

The chiropractic profession should openly embrace, and become actively involved in, established public health initiatives. The APHA is by far the largest and most influential public health organization in the United States. It wields tremendous influence on policy and procedure in our healthcare system. In 1983 a few chiropractic pioneers began what eventually became the Chiropractic Section of APHA [16]. This section is made up of dedicated individuals who care about promoting and taking part in APHA activities. Some examples of these activities are provided in Table 1. However, these dedicated individuals did this with very little support from the profession as a whole. Even now, very few chiropractic physicians are members of the APHA.

**Table 1:** Examples of activities of chiropractors within the American Public Health Association (APHA)

1. Chiropractic members of the APHA conducted a session on immunization in 1992, which was attended by several epidemiologists from the Centers for Disease Control.
2. A chiropractor served as Chair of the APHA Intersectional Council in 2000–2001.

3. A chiropractor served on the APHA Executive Board in 2000.

4. Several papers authored by chiropractors have been published in the Journal of the American Public Health Association.

5. A chiropractor organized and presided over a special session called "Faith, Terror, Hope, and Public Health: Exploring the Common Ground" shortly after 9/11.

6. In 2002 the Chiropractic Health Section won an APHA Intersectional Council grant to promote collaboration between sections. They teamed with the Vision Care, Podiatry, and Oral Health Sections to produce a mega-booth in the exhibit at the Annual Meeting, which was awarded 2nd place in 2002 and a tie for 1st place in 2003 for best exhibit.

7. In 2005, with the help of chiropractic members of the APHA, the American Chiropractic Association began including a public health column in its online publication.

8. A chiropractor introduced the Surgeon General of the United States in a special APHA session in 2002.

9. A chiropractor received a gold watch and award/recognition for recruiting more members than any single person in APHA's 125 year history.

10. A chiropractor serves on the APHA Forum on Aging.

One immediate action step that individual chiropractic physicians can make is to join and become active in the APHA. This would be one of the best ways for chiropractors to have an influence on public health policy. Spinal pain is an enormous public health issue, as the vast majority of Americans will develop a painful back or neck that will require treatment some time in their lives. Back pain-related conditions make up three of the top 10 conditions in the US, and the cost to society from spinal pain is amongst the highest for any condition [17-19]. Employers are looking for ways to prevent disability from low back pain on the job, and we could fill tremendous void in public health by providing educational programs to the public on how to prevent spinal pain and its related disability. This could provide exposure of chiropractors to a variety of segments of society (since all are affected by spinal pain), including athletes, the elderly, children, workers and military personnel.

It is also vital that those chiropractors who dogmatically oppose common public health practices, such as immunization [15] and public water fluoridation, cease such unfounded activity. In fact, because of the traditional chiropractic opposition of these well-accepted public health practices, there was major concern regarding whether chiropractic would even be accepted within the APHA [16]. In addition, the profession must take an honest public health-oriented approach to clinical practice and wellness care by becoming more involved in teaching patients how to stay healthy without frequent, endless visits to chiropractic offices. We are concerned that the common perception (which is well supported, in our experience) that chiropractors are only interested in "selling" a lifetime of chiropractic visits may be one of the primary factors behind our low standing in the minds of members of the public [2]. This is supported by a Canadian study which found that when the public was educated about "subluxation", the cornerstone of many chiropractors' "lifetime treatment plans", members of the public actually developed a negative view, and were more likely to want to consult a medical doctor to see if they had a subluxation prior to seeing a chiropractor [20]. The recommendation for repetitive life-long chiropractic treatment compromises any attempt at establishing a positive public health image and needs to change. Public health is ultimately about self-empowerment and teaching people how to take care of themselves, with an emphasis on prevention and health maintenance. The chiropractic profession should adopt the APHA's scientifically-grounded emphasis on nutrition and exercise as the "keys to wellness" (; accessed 3 June, 2008), as opposed to the common "lifetime adjustments" approach.

#### 2. Educational Reform

In 1961 podiatric medicine underwent its own version of allopathic medicine's Flexner Report. Known as the Selden Commission Report [21,22], it led to several improvements in podiatric medical education, some of which are similar to improvements that have been made to chiropractic education, including the adoption of identical requirements to those of all medical schools, advances in faculty development and major library expansion. In addition to these upgrades in the podiatric educational requirements, the Selden Commission report promoted the placement of podiatric education under the aegis of universities, with the inclusion of federally funded research [21]. This led to further movement of the podiatric medical profession toward integration within the healthcare system by mainstreaming its educational institutions as well as demonstrating, and providing support for, its commitment to research. Equally important, it led to the recognition of podiatric physicians as being on equal par with Medical Doctors, Doctors of Osteopathy and Dentists [22]. More recently, the podiatric medical profession has undergone an Educational Enhancement Project [23] in which the profession examined its educational process, from the point of acceptance to podiatry school to the point of becoming board certified. Comparisons to allopathic and osteopathic education were undergone to determine those areas in which podiatric education fell short. Changes were made to bring podiatric education up to par with that of these other professions.

According to the American Association of Colleges of Podiatric Medicine, there are eight accredited schools of podiatric medicine in the United States, with five of these programs (62.5%) based within a university setting. Having podiatric education integrated within a university setting brings a certain level of respect as a mainstream profession. The culture of University-based academics stresses the importance of scholarship amongst faculty as well as academic freedom. This allows for growth and change of the profession's knowledge base [24]. It also allows for interaction between the podiatry students and students of other disciplines, fostering integration and understanding about their unique specialty.

The chiropractic profession has at times made significant advances in classroom education and accreditation. Known as "Chiropractic's Abraham Flexner" [1], John J. Nugent, DC helped bring about a number of beneficial changes in chiropractic education, including increasing the number of years for chiropractic education, the conversion of chiropractic "trade schools" into non-profit professional institutions and the standardization of curricula. It must be noted that Nugent was despised by many within chiropractic, particularly BJ Palmer, because of his efforts [1]. In addition, even with the efforts of Nugent and others in bringing about improvement, chiropractic education still remains behind other health professions in a number of key areas, particularly those of clinical exposure of students to a variety of clinical situations [25] and involvement of faculty in the advancement of new knowledge in the field [26].

We feel that the profession must undergo its own version of the Flexner Report in medicine, and/or the Selden Commission Report and Educational Enhancement Project in podiatry. That is, we must take a critical look at our educational institutions, find what is substandard, and correct those deficiencies. One of the problems that we encounter frequently in our interaction with chiropractic educational institutions is the perpetuation of dogma and unfounded claims. Examples include the concept of spinal subluxation as the cause of a variety of internal diseases and the metaphysical, pseudo-religious idea of "innate intelligence" flowing through spinal nerves, with spinal subluxations impeding this flow. These concepts are lacking in a scientific foundation [27-29] and should not be permitted to be taught at our chiropractic institutions as part of the standard curriculum. Much of what is passed off as "chiropractic philosophy" is simply dogma [30], or untested (and, in some cases, untestable) theories [27] which have no place in an institution of higher learning, except perhaps in an historical context. Faculty members who hold to and teach these belief systems should be replaced by instructors who are knowledgeable in the evidence-based approach to spine care and have adequate critical thinking skills that they can pass on to students directly, as well as through teaching by example in the clinic.

In addition, chiropractic faculty should be required to engage in research and scholarship. Currently, the bulk of such activity in chiropractic educational institutions is carried out by just a few individuals, with a recent trend toward a falling publication rate [26]. In most other traditional university settings, including podiatric colleges, faculty are expected to "publish or perish". This level of academic excellence needs to permeate the chiropractic colleges as well.

Consideration should also be given to upgrading admission requirements to chiropractic schools. In podiatric medicine, such upgrading, which included the requirement of the Medical College Admission Test (MCAT), a requirement of medical school admission, is considered one of the significant events in the profession's history, giving the profession legitimacy in its calls for parity with medicine [21]. Lest there be concern amongst chiropractic colleges for diminishing enrollment if this type of upgrade were instituted, it should be noted that podiatric medicine experienced an increase in students following the institution of the MCAT requirement [21].

#### 3. Residency Programs in Hospitals

The podiatric medical profession began hospital-based postgraduate training in 1956 [31]. This training was officially sanctioned as a residency program in 1965 [31]. Important in the progress of residency training was when podiatric regulatory bodies started requiring residency training as a condition of licensure [31]. So the development and progression of residency training in podiatry was brought about not only by the academic portion of the profession, but also by the regulatory portion. This led not only to improved clinical competence of podiatrists, but also to greater respect for, and confidence in, podiatric physicians on the part other healthcare groups as well as by the public at large. Working within hospital-based residency programs allowed podiatrists to be considered peers of the medical community. This type of professional and cultural authority has its roots in the daily interaction between podiatric residents and the other medical physicians in these hospital-based residency programs.

It is essential that the chiropractic profession establish hospital-based residencies [25]. There is a tremendous void in how chiropractic graduates develop any meaningful hands-on clinical experience with real patients in real life situations. It is widely recognized in medical and podiatric education that abundant exposure to clinical environments is essential to developing top-quality professions. The Council on Chiropractic Education requirement of 250 adjustments forces interns to use manipulation on patients whether they need it or not, and the radiographic requirement forces interns to take radiographs on patients whether they need them or not. Rather than focus on interns meeting certain numerical requirements, interns should be encouraged to develop clinical decision making and patient management skills. Further, the emphasis on achieving a certain number of procedures as opposed to the acquisition of skill and knowledge impedes the development of professional moral reasoning by training interns to use patients as a means to meet their own goals, rather than focusing on the needs of the patients themselves.

The chiropractic internship should, as with medicine and podiatry, occur *after *graduation. Because chiropractic physicians are not trained in surgery, it may not have to last the full four years that many podiatry residencies entail [31], but we feel that the post-graduate internship should last a full year, with a second year of residency following the internship. The internship and residency should occur partly in a hospital, and partly in outpatient centers of excellence in which the intern/resident takes part in clinical decision making and patient management under the supervision of chiropractic physicians who are among the top in their field.

Chiropractic regulatory bodies such as state boards of chiropractic medicine should move in the direction of requiring the completion of postgraduate residency training as a condition of licensure. As was the case in podiatric medicine, this new requirement would force the profession to upgrade the training of its new practitioners to include a post-graduate residency.

#### 4. Clear Identity

Perhaps the most important factor that helped the podiatric medical profession to flourish was the fact that podiatrists had a clear identity and purpose; the podiatric medical profession was founded on the purpose of filling a need in society – the care of problems of the foot. They did not invent a "lesion" and a "philosophy" and try to force it on the public. They certainly did not claim that all disease arose from the foot, without any evidence to support this notion. The podiatric medical profession simply did what credible and authoritative professions do [32] – they provided society with services that people actually wanted and needed.

The podiatric medical profession focused on a particular set of problems for which allopathic medicine had little interest and a limited ability to deal with effectively, i.e., common foot disorders [6]. A key occurrence in the development of the podiatric profession was when the AMA determined that medical physicians should not get involved with "minor" foot problems. This opened the door for podiatrists to flourish in their chosen area of specialty, and retain complete control of their scope of practice without fear of intrusion by organized medicine [6]. The podiatric medical profession did not challenge the medical profession with claims of being an alternative method of treatment for medical problems.

The chiropractic profession must establish a clear identity and present this to society. In the beginning, DD Palmer invented a lesion, and a theory behind this lesion, and developed a profession of individuals who would become champions of that lesion. This is not what credible professions do. A credible profession is one that is established by society to meet a need that society itself has decided must be met [32]. Based on all the evidence regarding chiropractic practice and education, there is only one societal need (but it is a huge one) that chiropractic medicine has the potential to meet: non-surgical spine care. Our education and training is focused on the spine, and clearly if there is a common bond among all chiropractors, it is spine care [33]. While there are a variety of practitioners who offer spine care (physical therapists, osteopaths, movement specialists, massage therapists) there is no physician-level specialty that has carved a niche as society's one-and-only non-surgical spine specialist whose expertise is focused on the diagnosis and management of spine disorders.

We often hear from chiropractors that "chiropractic is more than just back pain". But is it? And, more importantly, does it have to be? Studies have demonstrated that yes, chiropractic is more than just back pain. It is back pain, neck pain and, occasionally, headache [34-36]. We feel that the primary reason the chiropractic profession has survived for 110+ years to the extent that it has is that manipulation is very helpful for many people with back and neck pain. Back pain, neck pain and headache are virtually the only reasons people consult chiropractors [34-36].

Some chiropractors reading this statement may be thinking, "This may apply to the rest of the profession, but my patients see me for wellness and a variety of visceral problems". We would ask these readers to look critically at this assumption. Hawk, et al [37] sought out practices that made that very claim, i.e., practices that claimed that a substantial percentage of their patients saw them for non-musculoskeletal complaints. They asked the patients the reason they were attending for treatment. Ninety percent of the patients stated that they were seeing the chiropractor for musculoskeletal problems. Recall that these were practices that were specifically sought out because they claimed to see a high percentage of non-musculoskeletal complaints. Before any chiropractor thinks of his or her practice as including a large number of non-musculoskeletal conditions, we suggest they ask their patients first. Or, better yet, have an independent source ask the patients. Chances are the reality will be much different than the perception.

No matter how one looks at it, or what one would like reality to be, chiropractic medicine is about back pain, neck pain and headache. Instead of fighting that fact (or denying it), we should embrace it fully and focus on becoming society's go-to profession for disorders in this area. First, spine-related pain is one of the largest markets in all of health care. Considering neck/arm pain, back/leg pain and headache, virtually 100% of the population is potentially included [38,39] (contrast this with the fact that only 7.5% of the population currently see a chiropractor [3]). Second, no medical specialty has successfully carved a niche for itself in this area (although the physical therapy profession is moving rapidly in this direction). Third, spine-related disorders create a great deal of suffering on the part of patients, in addition to exacting great costs on employers, the healthcare system and society at large. Providing much-needed high quality care to individuals suffering from spinal pain, as well as initiating and taking part in public health campaigns designed to educate people about spinal pain, would be a great service to society, and would bring millions of new patients to chiropractic offices, patients who would not ordinarily consider seeing a chiropractic physician.

The chiropractic profession fairly recently had a unique opportunity to catapult itself into the role of society's non-surgical spine specialists. In 1994 the Agency for Health Care Policy and Research released its guidelines on the management of acute low back pain in adults [40]. These guidelines recommended spinal manipulation as one of the only treatments for which adequate evidence existed for its efficacy. The report received a great deal of media coverage, with some media outlets actually mistakenly identifying "chiropractic", rather than "manipulation" as the recommended first-line approach. We could have used this as a springboard to moving ourselves into the mainstream as the premier non-surgical spine specialists in society. However, the profession did not jump at the chance, largely, in our experience, for fear of being "limited" by the image. Ironically, the profession chose to avoid being "limited" to the management of a group of disorders (back pain, neck pain and headache) that affect virtually 100% of the population through all stages of life [41]. In the interim it has seen its market share dwindle from 10% of the population [4] to 7.5% [3,42]. Even amongst patients with back pain, the proportion of patients seeing chiropractors dropped significantly between 1987 and 1997, a period of time in which the proportion seeing both medical doctors and physical therapists increased [43].

It is interesting that chiropractors have traditionally prided themselves on being "holistic". The emerging model of modern spine care is the "biopsychosocial" model [44]. That is, it is increasingly recognized that in order to provide optimum care for patients with spine-related disorders, one has to consider the *whole person*. Thus, non-surgical spine care provides chiropractic medicine with a wonderful opportunity to provide truly holistic care for patients, and to be recognized for expertise in this area. This would certainly be a drastic departure from the reductionistic subluxation-only approach, which "reduces" the cause and care of health problems to a spinal subluxation. Further, because the biopsychosocial approach often requires multidisciplinary involvement, embracing this model will further help to integrate chiropractic medicine into mainstream health care.

The World Federation of Chiropractic (WFC) has taken an important step in establishing a clear identity for chiropractors as "The spinal health care experts in the health care system" [45]. It is critical that other state, provincial and national associations follow the lead of the WFC.

#### 5. Fidelity to the Social Contract

The professions, which classically included medicine, law and the ministry, are vocations whose members "profess" to have knowledge that the laity do not comprehend. Given the asymmetry of knowledge between professionals and the laity, society has granted to the professions a certain degree of autonomous control over themselves. However, this social contract demands that each profession, and each professional, place the wellbeing of society and the patient, client or parishioner ahead of the profession and professional. Lay persons put their faith in the professional following the dictum *credat emptor *(let the buyer have faith) rather than *caveat emptor *(let the buyer beware) [32]. This social contract imparts great freedom on all professions, but with this freedom comes great responsibility.

When an individual consults a member of any of the medical professions, it is reasonably expected that the advice and treatment that he or she receives is based in science, not metaphysics or pseudoscience. In addition, it is reasonably expected that the services he or she receives are being provided for the primary purpose of benefiting the patient, and not for any other reason. The financial benefit to the professional is secondary, and results from the degree of clinical benefit received by the patient. Patients place their faith in the professional, and trust that they will not be subject to fraud, abuse or quackery. This is the social contract as it applies to chiropractic physicians.

By focusing on a specific set of clinical problems (i.e., foot disorders) for which society had a demonstrable need for professional services, using the scientific method to explore ways to better serve society, consistently upgrading their clinical training, and appropriately policing themselves, podiatrists have successfully fulfilled the social contract. As a result, it is our experience that podiatrists are widely perceived by the public to be ethical and honest professionals who generally have their patient's best interests at heart.

The chiropractic profession has an obligation to actively divorce itself from metaphysical explanations of health and disease as well as to actively regulate itself in refusing to tolerate fraud, abuse and quackery, which are more rampant in our profession than in other healthcare professions [46]. This must be done on an individual practitioner basis as well as by the political, educational and regulatory bodies. In this way the profession can fulfill its responsibility to the social contract. This will dramatically increase the level of trust in and respect for the profession from society at large.

#### 6. Podiatrists and Foot Reflexologists

We feel it is important here to briefly contrast and compare podiatry and foot reflexology. While the two professions have always been distinct, there is commonality in that each focuses its treatment efforts on the foot; however, this is where any resemblance between the two professions ends. Podiatric medicine is a science-based profession dedicated to the diagnosis and treatment of foot disorders. Foot reflexology is a metaphysically-based group consisting of non-physicians who believe that many physical disorders arise from the foot. Podiatrists have rejected foot reflexology as an unproven and unscientific practice, and do not consider it part of mainstream podiatric practice. Thus, it would be quite unreasonable to think that podiatry and foot reflexology could ever exist under one professional roof.

Yet, this is the very untenable situation in which we find ourselves in the chiropractic profession. Chiropractic has frequently been described as being two professions masquerading as one, and those two professions have attempted to live under one roof. One profession, the "subluxation-based" profession, occupies the same metaphysical and pseudoscientific space as foot reflexology. The other chiropractic profession – call it "chiropractic medicine" as we do in this commentary – has attempted to occupy the same scientific space as the podiatric profession. Alas, the marriage of convenience between these two chiropractic professions living under one roof has not worked. We find science-based practitioners and organizations alongside quasi-metaphysical, pseudoreligious, pseudoscientific practitioners and organizations. The result is continual battling with a huge waste of energy and resources, while professional growth stagnates.

We must finally come to the painful realization that the chiropractic concept of spinal subluxation as the cause of "dis-ease" within the human body is an untested hypothesis [27]. It is an albatross around our collective necks that impedes progress. There can be no unity between the majority of non-surgical spine specialist chiropractic physicians and the minority of chiropractors who espouse metaphysical, pseudoreligious views of spinal subluxations as "silent killers" [47]. The latter minority group needs to be marginalized from the mainstream majority group, and no longer should unrealistic efforts be made toward unification of these disparate factions within the profession.

## Conclusion

Reform of the chiropractic profession is long overdue. We need to make dramatic changes in the profession if we are to advance ourselves in the direction of becoming a credible, respected and widely utilized profession. Many mistakes were made in the past that prevented us from making this advancement. However, it is not too late to correct these mistakes. There is an example of a profession that, in the same 110+ years that the chiropractic profession has existed, has achieved the kind of mainstream acceptance that we have failed to achieve. We suggest that we examine how we may benefit from the experience of this other non-allopathic profession. The podiatric medical profession succeeded in establishing itself as a mainstream profession because of certain specific actions it took, and certain actions it did not take.

We see a tremendous opportunity for chiropractic medicine to become what it can and should be: a profession of non-surgical spine specialists who not only offer one useful modality of treatment for spinal pain (manipulation), but offer something much greater and more important – expertise in the diagnosis and management of spinal pain patients. This includes understanding the vast mechanisms of spinal pain as well as diagnosis, treatment and coordination of the treatment of other members of the healthcare team. It also means mastering a variety of non-surgical methods other than just manipulation that are useful in the management of patients with spinal pain. But, most importantly, it means becoming experts in *patient management*, i.e., helping patients overcome spinal pain, whether that means providing adjustments, exercise, short-term medication use and/or education regarding the issues related to LBP provided in a cognitive-behavioral context. Currently, there is no profession that adequately fills that role, although as we noted earlier, the physical therapy profession is moving quickly in this direction. The opportunity is there for us to correct our mistakes, but we must act now. The only question is whether the chiropractic profession has the integrity, vision and self reflection required to make the necessary changes. Time will tell.

## Competing interests

Each of the authors makes his living practicing, teaching, administrating or studying chiropractic medicine (or some combination of these activities) and thus has a financial interest in the success of the profession.

## Authors' contributions

DRM originally conceived of the conceptual basis of the paper and had detailed discussions of this with MJS, DRS, SMP and CFN both in person and via e mail. DRM then wrote the initial manuscript and this was distributed multiple times between MJS, DRS, SMP and CFN until the final manuscript was created. All authors took part in editing and revising the manuscript on multiple occasions.
